# Parenting Interventions for Child Mental Health: The Science and Practice of Measurement-Based Care

**DOI:** 10.1007/s10567-026-00561-x

**Published:** 2026-04-16

**Authors:** Adrienne I Turnell, David J Hawes, Vilas Sawrikar, Mark R Dadds, Lucy A Tully

**Affiliations:** School of Psychology, University of Sydney, Sydney, NSW Australia

**Keywords:** Measurement-based care, Routine outcome monitoring, Feedback, Parenting interventions, Child mental health

## Abstract

Evidence-based parenting interventions (EBPIs) are among the most powerful interventions in child mental health, but they are often impacted by non-response and drop-out. Extensive research has demonstrated that measurement-based care (MBC), which involves routine collection of client-reported measures to track intervention progress and inform shared decision-making, is associated with improved outcomes in adult mental health populations, including reduced rates of deterioration and drop-out. Comparatively, there has been little research on MBC in child mental health. MBC is inherently more complex and nuanced in EBPIs for child mental health due to the need to collect and integrate data from multiple caregivers, therefore the application of MBC requires careful consideration. This paper presents key considerations and recommendations for the effective implementation of MBC in EBPIs, with a focus on the application of the *Collect*,* Share*,* Act* Model (Barber & Resnick, 2022). Key considerations and recommendations are provided for: (1) collecting multi-informant data, (2) selecting measures, (3) administering measures, (4) sharing and integrating multiple and discrepant perspectives, (5) examining trajectories of change in EBPIs, (6) using MBC data to optimise clinical decision making and intervention planning, (7) considering organisational factors that may affect implementation, and (8) using MBC with culturally and linguistically diverse populations. A new framework is provided, the *Parenting CIRCLE of MBC*, to guide how MBC data can inform clinical decision making to personalise EBPIs to the needs and preferences of families.

## Introduction

Mental health problems emerge early in life with 50% of mental health problems emerging before the age of 14 years (Kessler et al., 2005), and are associated with a range of physical and mental health problems in adulthood (Caspi et al., 2020; Mulraney et al., 2021). Quality of parenting and parent-child relationships are important risk and protective factors for mental health in children. For example, children are at risk of developing emotional and behavioural problems when exposed to unpredictable and conflictual family environments and parent-child relationships characterised by low warmth and responsiveness, and a high level of coercion and hostility (Aitken et al., 2023; Felitti et al., 1998; Pinquart, 2017a, b). On the contrary, there is good evidence that interventions that target early child-rearing environments, such as evidence-based parenting interventions (EBPIs), are effective in reducing child mental health problems across diverse cultural populations and service contexts (Backhaus et al., 2023; Gardener et al., 2016). EBPIs are structured skills-based interventions that target parents/caregivers’ (hereafter referred to as ‘parents’) of children in early-to-middle-childhood (2–12 years) and aim to build knowledge and skills that support child development, strengthen parent-child relationships, and improve children’s emotional and behavioural outcomes (Barlow & Coren, 2018; Backhaus et al., 2023). Although underpinned by various theoretical orientations (e.g., attachment, mindfulness, cognitive behavioural principles), the most prominent and well-supported EBPIs are based on social learning theory and focus on improving child externalising behaviours (e.g., aggression, non-compliance, and oppositional behaviours), such as Parent-Child Interaction Therapy (PCIT; Eyberg et al., 1995), Triple P-Parenting Program (Sanders, 1999), and Incredible Years (Webster-Stratton, 2011).

Despite the wealth of evidence supporting the efficacy of EBPIs for child mental health, they are not effective for all children and families, rates of attendance are highly variable, and rates of intervention non-response and drop-out are high (Chacko et al., 2016; Ollendick et al., 2016; Shelleby & Shaw, 2014). Additionally, a review of interventions for child mental health has found mean effects have not improved measurably over the past 50 years, and for some outcomes such as conduct problems effect sizes have decreased, suggesting that interventions are becoming less effective over time (Weisz et al., 2019). As such, new intervention approaches are needed to improve engagement and outcomes for EBPIs for child mental health.

One promising approach is Measurement-Based Care (MBC), which involves systematically and routinely collecting client-reported measures and feedback to track progress, share results with clients, and use the data to guide shared decisions about the intervention (Scott & Lewis, 2015). Compared with assessment frequencies typically used in clinical practice (Tully et al., 2024), such as pre- and post-treatment assessment, the cornerstone of MBC is *regular* assessment (typically session-by-session) in the context of ongoing treatment for the purpose of monitoring treatment progress and process. MBC creates a mechanism for continuous feedback loops between practitioners and clients that may alert the practitioner to meaningful changes in intervention trajectory, such as a worsening of outcomes, and lead to timely and responsive adjustments to interventions, where appropriate (Tam & Ronan, 2017). For example, MBC can guide decisions about case conceptualisation, treatment approach, treatment format, and termination or discharge planning. Research has shown that practitioners tend to overestimate their effectiveness and have difficulty predicting client deterioration and drop-out (Hannan et al., 2005; Hatfield et al., 2010; Østergård et al., 2024; Walfish et al., 2012), so MBC can mitigate these practitioner biases and complement clinical decision-making to improve client engagement and outcomes. MBC represents the intersection between standardisation and personalisation of mental health care, such that MBC becomes a vehicle to standardise client feedback about intervention progress, and a process to promote shared decision-making and *dynamically* tailored interventions to client needs and preferences. While evidence for the efficacy of MBC has been demonstrated in adult mental health (Rognstad et al., 2023), limited research has been conducted in child mental health (Bergman et al., 2018; Tam & Ronan, 2017), and no research has examined the efficacy of MBC in EBPIs for child mental health problems.

EBPIs are nuanced mental health interventions because, although the outcomes of interest are the child’s emotional and behavioural wellbeing, the recipient of the intervention is parents, and the focus of the intervention is on the family system. Change processes are multifaceted with change occurring across various individuals in the family system, not just the individual/s attending sessions (e.g., change might occur in the parent, child, or parent-child system, partner system, or even broader family and sibling systems), which makes the use of MBC data to guide intervention planning complicated. Given the nuances of EBPIs, it is reasonable to anticipate that integration of MBC may present unique challenges and complexity, including the need to collect and integrate data from multiple parent participants; incorporate measures that are clinically meaningful and representative of change processes in EBPIs; identify trajectories of progress in EBPIs and whether cases are considered “off track” for positive progress; and operationalise feedback conversations with multiple parents to guide intervention planning. Considering the complexity of EBPIs, guidance is needed for parenting practitioners on how to best integrate MBC into existing intervention protocols. To our knowledge, the application and operationalisation of MBC in EBPIs has not yet been considered in the MBC literature.

Models and “best practice” guidelines (Boswell et al., 2023; Brooks-Holliday et al., 2021) to inform the operationalisation of MBC in clinical practice have started to emerge in the literature, with the *“Collect Share Act Model”* (Barber & Resnick, 2022) as one of the most comprehensive to date. However, these models are generic in nature and focused on adults in individual therapy, which can limit their utility for other populations and settings. Importantly, it should not be assumed that all clinical contexts and populations are the same as research demonstrates that MBC does not confer benefits with all client populations, such as populations with personality disorders (de Jong et al., 2018) and severe psychopathology (Errázuriz & Zilcha-Mano, 2018). Therefore, careful consideration is required as to how MBC principles apply to diverse and specialised populations, such as EBPIs for child mental health. Given low adoption rates of MBC among both child and adult mental health practitioners (Ionita & Fitzpatrick, 2014; Jensen-Doss et al., 2018, 2025; Cho et al., 2021; Tully et al., 2024), it is important that MBC is adequately tailored for EBPIs to ensure uptake by practitioners and the overall sustainability of implementation. A lack of adequate training and support have been consistently identified as barriers to MBC implementation in both adult (Lewis et al., 2019) and youth populations (Whitmyre et al., 2024), highlighting that a clear challenge for MBC implementation is for practitioners to have sufficient knowledge and skill about how to use MBC data in their clinical work (Mackrill et al., 2019). As such, it has been recommended that formalised training, clinical supports, and MBC guidelines are needed to support the implementation of MBC (Boswell et al., 2023; Kwan et al., 2021; Mackrill et al., 2019; Whitmyre et al., 2024). To the best of our knowledge, these supports do not yet exist for MBC implementation in EBPIs. Specific models of the application of MBC in EBPIs are also needed prior to research examining the efficacy of MBC in EBPIs.

The aim of this paper is to present key considerations and recommendations for the effective implementation of MBC in EBPIs, with a focus on the application of the *Collect*,* Share*,* Act* Model (Barber & Resnick, 2022). Key challenges with the operationalisation of MBC in EBPIs are discussed with consideration and recommendations provided for: (1) collecting multi-informant data, (2) selecting measures, (3) administering measures, (4) sharing and integrating multiple and discrepant perspectives, (5) examining trajectories of change in EBPIs, (6) using MBC data to optimise clinical decision making and intervention planning, (7) organisational factors that may affect implementation, and (8) using MBC with culturally and linguistically diverse populations. A new framework is provided, the *Parenting CIRCLE of MBC*, which offers a step-by-step guide of how MBC data can inform clinical decision making to personalise EBPIs to the needs and preferences of families.

## Measurement-Based Care in EBPIs: Current Evidence and Future Directions

There has been extensive meta-analytic research to support the efficacy of MBC in adult populations (de Jong et al., 2021; Lambert et al., 2018; Østergård et al., 2020; Rognstad et al., 2023; Shimokawa et al., 2010). Compared to standard monitoring, MBC has been associated with small but robust effect sizes in improving mental health outcomes, with effect sizes differing across the type of feedback system used (*d* = 0.14–0.40; Lambert et al., 2018; Rognstad et al., 2023). MBC has also been associated with reduced drop-out and deterioration rates (de Jong et al., 2021; Lambert et al., 2018), faster and better diagnostic procedures and improved communication between practitioners and clients (Carlier et al., 2012), and improved cost-effectiveness of mental health interventions (Delgadillo et al., 2021). MBC has shown stronger effect sizes (*d* = 0.29–0.36) in improving early detection and mental health outcomes in adult populations at risk of non-response and intervention failure (i.e., “not-on-track (NOT)” populations; Lambert et al., 2018; Shimokawa et al., 2010; Rognstad et al., 2023), with the strongest effect sizes (*d* = 0.36–0.49) demonstrated in MBC that includes clinical support tools that provide structured and formalised problem-solving recommendations for NOT cases (de Jong et al., 2021; Lambert et al., 2018). The modest effects of MBC should not be underestimated as the overall impact of MBC translates to an average 8% advantage over existing evidence-based mental health interventions, with benefits being even higher when used with clinical support tools (Barkham et al., 2023). These modest effects might also be an under-representation of the true impact of MBC as many studies report low adoption rates of MBC by study clinicians, and MBC is often not delivered as intended within studies (de Jong et al., 2021; Rognstad et al., 2023). Although MBC has demonstrated efficacy in adult populations, its effectiveness in child and youth mental health remains comparatively under-researched, with less than 10% of studies on the efficacy of MBC being conducted in child and adolescent populations (de Jong et al., 2021).

To date, four reviews have been conducted on the efficacy of MBC in youth populations. First, recent meta-analytic evidence has found that MBC has been associated with significantly larger effects in child and adolescent studies (Cohen’s *d* = 0.29) compared to adult studies (*d* = 0.12), suggesting that youth may benefit more than adult populations from MBC (Rognstad et al., 2023). Only individual psychological interventions targeting “common mental health disorders” (e.g., depression, anxiety) were included in this meta-analysis, and of the 31 RCT studies included, only four studies explored MBC in youth populations, so these results should be interpreted with caution.

Second, a meta-analytic review of nine studies (10 trials; *N* = 3804) demonstrated that MBC was associated with improved symptom severity, functioning, and goal attainment for youth aged 10 to 19 years receiving psychological interventions, with effect sizes in the small range (*d* = 0.28; Tam & Ronan, 2017). Psychological interventions were predominantly delivered to children and adolescents in individual or group format and included various therapeutic approaches (e.g., cognitive-behavioural, psychodynamic, family systems, eclectic) for a wide variety of mental health problems (e.g., emotional and behavioural problems, substance abuse). Only one study included parents as recipients of both MBC and the psychological intervention, which was a single case design approach with four families that explored the effects of family-centred feedback-informed therapy (FC-CFT) for youth aged 10–15 years with a diagnosis of conduct disorder (Ronan et al., 2016).

Third, Bergman and colleagues (2018) conducted a Cochrane review on the impact of MBC on psychotherapy outcomes for youth aged 11–18 years (six trials; *N* = 1097; most studies overlapped with Tam & Ronan [2017]) and found insufficient evidence to conclude MBC improved youth outcomes. Again, studies included various therapeutic approaches for a range of mental health concerns; however, none of the studies included an EBPI. Finally, in a narrative review, Parikh and colleagues 2020 identified 14 published studies examining the effectiveness of MBC in youth populations using a variety of experimental and non-experimental studies. Parikh et al 2020 concluded that the evidence-base demonstrates support for the use of MBC in child and adolescent populations in individual therapy conducted in school and outpatient settings, but not in group therapy formats. Notably, Parikh et al 2020 reported that MBC has been associated with reductions in youth mental health symptoms and psychological distress, as well as improvements in therapeutic alliance, and practitioner attunement and responsiveness to youth concerns. Importantly, the evidence-base for the use of MBC in youth populations is limited by a small number of heterogeneous studies, often with methodological limitations and inconsistent reporting on outcome measures (Bergman et al., 2018 ; Tam & Ronan, 2017; Rognstad et al., 2023). Despite these inconsistencies, there is general consensus among the literature that MBC demonstrates promise in youth populations aged 10–18 years and further high-quality research is needed, particularly studies exploring MBC with youth under 10 years (Bergman et al., 2018; Tam & Ronan, 2017). This is an important gap as interventions in early childhood often include parents, since they are the key agent of change.

For children under 10 years, MBC has predominantly been studied in individual child therapy, with studies with weak designs and variable methodological quality. In one of the only published RCTs, MBC was associated with significantly greater reductions in parent-reported total child distress compared to no MBC, among children aged 7–11 years (*N* = 38) attending play-based school counselling in the UK, with small to moderate effect sizes (Cooper et al., 2021). Notably, parents were not involved in the treatment. To date, no research has examined the efficacy of MBC in EBPIs for children under 10 years. Clearly, a priority for future research is to conduct high quality efficacy trials to examine whether MBC is associated with improvements in parental engagement and outcomes relative to no MBC. However, prior to conducting efficacy trials of MBC in EBPI, it is important to understand the nature and characteristics of EBPIs and how MBC can be effectively integrated into these interventions.

## Advancing Measurement-Based Care in EBPIs

Conceptually, MBC aligns well with the principles of EBPIs as consistent self-monitoring and independent problem solving are core skills acquired through EBPIs and are often considered key factors to intervention success (Sanders et al., 2019; Yu-Lefler et al., 2022). For example, parents are taught to observe and track children’s behaviours over time to build awareness and understanding of patterns of behaviours (e.g., common triggers, parent responses) and to measure change as parents implement new strategies. Session-by-session monitoring in MBC may be a means to formalise and make the process of self-monitoring concrete and standardised, while explicitly encouraging feedback conversations about intervention progress with parents. Additionally, many EBPIs tend to be relatively brief and delivered over 8–12 sessions (Barlow & Coren, 2018) and larger effects have been found for MBC in short-term therapies (e.g., under 3 months; Rognstad et al., 2023). This suggests that MBC could act as a vehicle for rapidly establishing therapeutic alliance in EBPIs as it incorporates the client’s perception and provides a shared understanding early on in an intervention (Rognstad et al., 2023), so MBC may be well-suited to brief EBPIs.

While research has demonstrated consensus among expert practitioners that use of measures for progress monitoring is a core practitioner competency for delivering EBPIs (Barker & Hawes, 2024), routine outcome measures are rarely adopted by practitioners working in child and youth mental health (Tully et al., 2024). Pre-to-post intervention assessments are commonly used within EBPIs to support diagnostic decisions and evaluate outcomes, and most EBPI models incorporate targeted pre-treatment assessments to guide decisions about treatment approach and to tailor interventions to individual family characteristics. For example, the Family Check-Up model (Dishion & Stormshak, 2007) utilises a comprehensive pre-treatment assessment and feedback session to improve treatment engagement by building family motivation for change and identifying a range of tailored family-based intervention options. The PersIn approach in “MY PCIT” (McCabe et al., 2020) also uses a targeted pre-treatment assessment to tailor EBPIs to be relevant and acceptable for ethnically and culturally diverse families. Although these assessment-driven approaches aim to improve the fit of EBPIs to a family’s characteristics, needs, preferences, and context, this type of assessment-driven practice is predominantly focused on initial treatment planning and does not provide the opportunity for ongoing collaborative decision-making and adjustments throughout interventions that are responsive to the needs of families. MBC extends beyond these approaches as it can be used to identify families at risk of non-response and deterioration throughout the intervention, and to guide decisions about whether, when, and how to modify an intervention flexibly and dynamically, and track outcomes associated with intervention modifications as they are implemented. Thus, MBC provides a standardised practice for EBPIs to be intentionally and responsively tailored to the needs and preferences of families throughout the intervention, with the aim of reducing rates of deterioration and drop-out, and improving outcomes.

EBPIs can be delivered in both individual parent/family-based formats or group formats (Backhaus et al., 2023). Evidence on the efficacy of MBC in group psychotherapy remains limited (de Jong et al., 2021) and findings on the efficacy of MBC in this format are mixed in adult and youth settings (Davies et al., 2008; Haug et al., 2025 ; Slone et al., 2015 ; Parikh et al., 2020). The use of MBC in group EBPIs brings its own set of complexities, particularly in terms of how group facilitator/s share and address individual parent feedback, whether they have capacity to adjust group interventions to accommodate individual feedback, and how they might manage ruptures in practitioner-parent alliances, as well as group alliances (Rognstad et al., 2023). A full discussion of the use of MBC in group settings is beyond the scope of this paper. As such, this paper will focus on MBC implemented within individual formats, which is where the evidence-base is most robust.

Integration of MBC into EBPIs will require nuanced consideration and guidance; however, no guidelines or models on the use of MBC in EBPIs are currently available. Although not grounded in the MBC literature, one of the best examples in the field of EBPIs for how to integrate MBC practices is PCIT. PCIT utilises session-by-session measurement to track client progress and guide intervention planning (McNeil & Hembree-Kigin, 2010), although it differs from MBC in several ways. Some key differences are that PCIT decisions are often protocol- or practitioner-driven (e.g., decisions of which skills to focus on each session are based on progress toward “mastery criteria”, McNeil & Hembree-Kigin, 2010) rather than a shared decision-making process between practitioners and clients that is fundamental to the practice of MBC; feedback is delivered to parents rather than arrived at through self-reflective practice and collaborative discussion; outcome measures are predominantly progress focused (e.g., symptom reduction, skill acquisition) rather than process focused (e.g., therapeutic alliance, engagement, motivation); progress measures are standardised for all families rather than personalised (e.g., parent goals); and, there is less scope to modify the intervention approach based on collaborative feedback discussions, thereby limiting the full potential of MBC. Although there is room for improvement, the inherently data-driven approach of the PCIT protocol demonstrates that MBC can be feasibly integrated within existing EBPIs. However, PCIT is a highly intensive intervention that follows an in vivo coaching format, so it is different from most EBPIs that rely on role-play and behavioural rehearsal and do not have the child present in session, so the model may not be easily generalisable to other EBPIs. In fact, research has demonstrated that PCIT practitioners’ use of routine outcome measures is low when they use therapeutic modalities other than PCIT (Klein et al., 2022). Practitioners reported a key difference between PCIT and other therapy modalities was that measures are integrated into the PCIT protocol, with guidance on how to use measures to inform intervention planning (Klein et al., 2022). This highlights that the practice of MBC is not easily generalisable across interventions, and practitioners have difficulty integrating and applying MBC practices to other EBPIs. Instead of prescribing an MBC protocol for a single EBPI, practitioners may benefit from guidance about key considerations and flexible recommendations about the integration of MBC principles across EBPIs. The *Collect*,* Share*,* Act* Model (Barber & Resnick, 2022) offers a strong foundation for this approach.

## The Collect, Share, Act Model: Key Considerations for EBPIs

The *Collect*,* Share*,* Act* Model proposes that MBC contains three core sequential phases, including: (1) routinely collecting client-reported outcome measures; (2) sharing timely feedback with the client about intervention progress; and (3) acting on feedback to make collaborative decisions about the course of the intervention (Barber & Resnick, 2022). Although these elements may appear straightforward, effective implementation in EBPIs requires thoughtful consideration and adaptation of each phase to the specific intervention characteristics and the demands of local implementation contexts. The following sections will explore relevant considerations and challenges of implementing each phase of this model within EBPIs.

### Phase 1: Collect

Three key issues have been identified with the application of the *Collect* phase to EBPIs, including collecting data from multiple participants, selecting valid and clinically useful measures that capture relevant change processes in EBPIs, and the availability of technological systems to administer and display MBC data for EBPIs.

#### Key Consideration 1: Collecting Multi-Informant Data

As contemporary child rearing is often shared by multiple caregivers, practitioners must first consider how many caregivers are invited to participate in the EBPI. Research suggests best practice involves the “core parenting team”, with evidence indicating parenting interventions involving both parents are more effective than mother-only interventions (Lundahl et al., 2008). Practitioners should come to a collaborative decision with the family about who will form the “core parenting team”. Once established, the team must then consider which caregivers are involved in the MBC feedback process. There are several ways to use MBC with more than one caregiver, with each one increasing in complexity.

First, and in line with how MBC has traditionally been implemented in individual therapy, practitioners may choose to have a single caregiver complete the measures. This is likely to be the primary caregiver, and while this option allows for easiest completion and interpretation of the outcome measures, it will exclude and undervalue the perspectives of other key caregivers, thereby limiting data available and reducing the utility of MBC. Second, practitioners may recommend that key caregivers complete one set of measures together by discussing and providing feedback based on a consensus, although this may be challenging where caregivers are discordant in their views, which can be common in EBPIs (Tully et al., 2021). Third, practitioners may recommend that caregivers complete the measures separately. Of these options, we recommend the third approach of collecting, sharing and acting on feedback from all participating parents as this approach communicates that all caregivers’ perspectives are critical to the intervention process, despite the complexities associated with addressing and balancing multiple perspectives. The reason for this recommendation is that, should the focus only be on the primary caregiver, for many families this will mean gathering data only from the mother, and the message that may be conveyed inadvertently is that fathers are less important to the intervention process than mothers (Tully et al., 2021). In fact, the use of MBC potentially offers a new strategy for enhancing the engagement of fathers in EBPIs and tracking their outcomes.

There is consistent evidence that levels of father involvement in interventions for child mental health are low (Dadds et al., 2018), and a recent systematic review of EBPIs found 58% of studies either did not report father engagement or had father engagement rates below 50% (Gonzalez et al., 2023). When an EBPI focusses on mothers as the sole agents of change, it is likely that risk and protective factors may inadvertently be modified in only one part of the parental system, thereby potentially reducing the effectiveness of the intervention (Tully et al., 2021). While use of MBC will not help increase father attendance, it may help engagement, prevent drop out, and improve father outcomes from EBPIs, especially as meta-analytic evidence suggests effect sizes for changes in fathers’ ratings of parenting and child behaviour in EBPIs are smaller when compared to mothers (Lundahl et al., 2008). MBC might function as a novel vehicle to provide fathers a ‘voice’ and sense of agency in EBPIs, subsequently supporting fathers to feel included and valued, while also supporting practitioners to value fathers’ perspectives. Overall, decisions about parent measure completion should be made together with caregivers, establishing a foundation for a truly collaborative MBC process.

In addition to the core parenting team, practitioners might consider the inclusion of other relevant informants in the MBC process (e.g., children, educators, other health professionals, the practitioner themselves). Multi-informant assessments are considered a gold-standard evidence-based approach for triangulating information in youth interventions, particularly in relation to diagnostic clarity and treatment outcomes (Hunsley & Mash, 2007). Incorporating data from multiple sources might offer a more comprehensive understanding of progress, especially regarding the generalisability of intervention effects across settings such as pre-school and school. MBC also offers an opportunity to integrate child perspectives, particularly in intervention models where children are active participants (e.g., PCIT, Collaborative and Proactive Solutions [Greene & Winkler, 2019], Cool Kids [Rapee et al., 2019]) and in cases where the child’s internal experience is central (e.g., internalising concerns). Limiting MBC to parent input risks aligning interventions solely with caregiver expectations, potentially overlooking the child’s needs (Bergman et al., 2018). Notably, discrepancies between youth and parent reports are common (De Los Reyes et al., 2022) and given that children rarely initiate mental health interventions, their goals may diverge from those of their caregivers. Including the child’s perspective, therefore, might add meaningful and clinically useful information for intervention planning. Of course, the feasibility of incorporating children in MBC would depend on several factors, including the child’s level of involvement in the intervention, age, cognitive ability, and suitability of the measures incorporated. Nonetheless, the inclusion of multiple informants requires careful consideration. Practitioners must weigh the added clinical utility against practical constraints such as time burden and resource availability, determine which informants are best positioned to contribute meaningful data and how to manage discrepancies between informants, and establish appropriate time frames for data collection (e.g., weekly, monthly). These decisions should be guided by sound clinical judgement and tailored to the specific context of each case.

#### Key Consideration 2: Selecting Measures

The measures chosen for MBC are a central implementation factor as negative client and practitioner attitudes toward measures, particularly perceptions that standardised measures are inferior to clinical judgement and do not capture meaningful change processes in therapy, are barriers to implementation (Lewis et al., 2019; Solstad et al., 2019). Given the complex and multifaceted change processes in EBPIs, a key consideration for parenting practitioners is including measures that have clinical utility and best capture these change processes, as well as measuring constructs known to impact response to EBPIs.

To date, MBC studies have predominantly focused on the impact of MBC on symptom reduction through “nomothetic” approaches to outcome monitoring, which includes the use of standardised measures normed across populations with strong psychometric properties relating to validity, reliability, and sensitivity to change (Scott & Lewis, 2015). However, research in adults has demonstrated larger effect sizes for measures of quality-of-life and functioning (d = 0.24) and therapeutic alliance (d = 0.28) than mental health symptoms (d = 0.11) (Rognstad et al., 2023), highlighting the importance of monitoring constructs reflective of client engagement and recovery beyond symptom severity. Additionally, concerns have been raised about the validity of nomothetic approaches for culturally diverse populations (Jacob & Edbrooke-Childs, 2022). As such, research has started to incorporate “idiographic” approaches, which involve a standardised questionnaire format but the items, and progress toward those items, are generated by the client and subsequently tailored to the needs and preferences of clients (Lloyd et al., 2019). There is emerging evidence for the reliability, validity, and clinical utility of idiographic measures in adult populations (Ashworth et al., 2009; Sales & Alves, 2016; Lloyd et al., 2019) and youth populations (Duncan et al., 2023; Weisz et al., 2011), with studies demonstrating that idiographic measures capture important information not otherwise obtained by standardised measures (Sales et al., 2018). Critically, qualitative research shows that clients support this shift in MBC, expressing that current measures often focus too narrowly on symptoms and overlook personal goals, values and broader life experiences, with clients advocating for measures that also address therapeutic alliance, family, social life, and daily functioning (Solstad et al., 2019). Therefore, including standardised nomothetic and idiographic measures might provide complementary indicators of change providing both normed comparative results, but also client-centred items that are representative of the individuals’ perception of meaningful change (Sales et al., 2023). Since the benefit of MBC depends on the clinical utility of the feedback data, the choice of routine measures used is of utmost importance (de Jong et al., 2023).

For EBPIs, it is recommended that measure selection in MBC be a shared decision-making process between the practitioner and core parenting team. Qualitative research indicates that clients value being involved in defining their outcomes, and that collaboration around the content and use of measures is important for successful clinical use of MBC (Solstad et al., 2019). Therefore, the measures selected should reflect parent preference, practitioner expertise, and the most relevant factors identified from a comprehensive clinical assessment. Practitioner judgement is a critical part of decision making as practitioners need to balance feasibility and burden of measures with what measures provide meaningful data for intervention planning, as well as what factors are modifiable and important to target in EBPIs (Barber & Resnick, 2022). Table 1 provides examples for a range of progress and process constructs that practitioners might consider measuring in EBPIs, including: child and parent mental health symptoms, quality of life and functioning, goal attainment, skill acquisition and implementation of strategies, co-parenting relationship, and therapeutic alliance.

**Table 1:** Example of measure constructs and instruments for use in MBC within EBPIs

Construct	Measurement approach	Example instruments
Child mental health symptoms	• Monitor single symptom groups over time (e.g., externalising or internalising problems), using standardised measures. • Monitor comorbid symptom groups over time (e.g., internalising, externalising, attention difficulties), using standardised measures. • Monitor specific parent-identified problems of concern over time, using idiographic measures.	• 36-item Eyberg Child Behaviour Inventory for children 2–16 years (ECBI; Eyberg & Pincus, 1999) or 9-item Weekly Assessment of Child Behavior-Positive Form for children 2–12 years (WACB-P; Timmer et al., 2021) for measuring externalising symptoms, or 38-item Spence Children’s Anxiety Scale for children 7–13 years (Spence, 1998) for measuring internalising symptoms. • 17-item Paediatric Symptom Checklist-17 for children aged 4–17 years (PSC-17; Gardner et al., 1999), or 25-item Strengths and Difficulties Questionnaire for children aged 2–17 years (SDQ; Goodman, 1997) for screening multiples symptom groups. • 3-item Caregiver Version of the Youth Top Problems (Weisz et al., 2011) for measuring the severity of the top three concerns/problems identified by the parent/caregiver (e.g., not listening to parent instructions). This idiographic measure is designed to complement standardised assessments of youth mental health symptoms by prioritising problems and adding specificity about identified problems (Weisz et al., 2011).
Quality of life & functioning	• Monitor level of interference or impact of child mental health problems over time, using standardised measures. • Monitor general functioning of the child over time, using practitioner judgment. • Monitor child health-related quality of life over time, using standardised measures.	• The SDQ (Goodman, 1997) includes additional questions that explore degree of distress, impairment, and burden associated with child mental health difficulties. • The Children’s Global Assessment Scale (CGAS; Shaffer et al., 1983) is a practitioner-rated measure of general functioning for children aged 4–16 years. • The parent version of the10-item KIDSCREEN-10 (Ravens-Sieberer et al., 2010) for general health-related quality of life, including subjective health and the psychological, mental and social wellbeing of children aged 8–18 years.
Goals	• Monitor progress toward parent goals throughout the EBPI, using idiographic measures.	• The Goal Based Outcomes tool (GBO; Law & Jacob, 2015) may be used to track progress toward the top three goals that have been identified for the EBPI. Goals might focus on child-based outcomes (e.g., reduction in aggression) and/or parenting outcomes (e.g., increased quality time spent with child).
Skill acquisition or implementation of strategies	• Monitor parent implementation of strategies between sessions, using unstandardised measures of homework completion. • Monitor parent acquisition of EBPI skills, using behavioural observation.	• Parent completion of homework, such as frequency of homework completion between sessions (e.g., number of days out of 7 for weekly sessions), could be visually graphed. This might aid parents to perceive the link between implementation of strategies and skill/intervention progress (McNeil & Hembree-Kigin, 2010). • Behavioural observation coding schemes, such as Dyadic Parent Interaction Coding Schedule (DPICS; Eyberg et al., 2013), may be used to track specific parent skills by observing and coding parent-child interactions in session. Use will depend on format of EBPI and whether the child is attending the session/s. Such coding schemes will likely require training and may be resource intensive.
Parent mental health	• Monitor parent general psychological distress over time, using standardised measures. • Monitor specific parent mental health symptoms over time, using standardised measures.	• The 10-item Kessler Psychological Distress Scale (K10; Andrews et al., 2001); or the 21-item Depression, Anxiety, Stress Scales (DASS-21; Lovibond & Lovibond, 1995). Such measures can also be used to monitor parent need for a mental health intervention throughout an EBPI. • The 9-item Patient Health Questionnaire – Depression (PHQ-9; Kroenke et al., 2001) for monitoring symptoms of depression; or 7-item Generalised Anxiety Disorder Assessment (GAD-7; Spitzer et al., 2006) for monitoring symptoms of anxiety.
Co-parenting relationship	• Monitor marital conflict specific to child-rearing over time, using standardised measures.	• The 16-item Parent Problem Checklist (PPC; Dadds & Powell, 1991) for a measure of parent conflict related to parents’ ability to agree and cooperate when performing parenting duties within their family.
Therapeutic alliance	• Monitor therapeutic alliance between parent and practitioner over time, using standardised measures.	• The 4-item visual analogue Session Rating Scale (SRS; Johnson et al., 2000), for parent perceptions of respect and understanding, relevance of the goals and topics, client-practitioner fit and overall alliance; or the 5-item Agnew Relationship Measure – 5 (ARM-5; Cahill et al., 2012) for parent perceptions of the bond, partnership and practitioner confidence in therapy. • The 12-item Therapeutic Alliance Scale for Caregivers and Parents (TASCP; Accurso et al., 2013) for measuring caregiver- and therapist-report of the caregiver-practitioner alliance in caregivers of children aged 4–13 years.

*Note:* *In the absence of available measurement feedback systems, practitioners will need to consider which measures are most useful to indicate reliable change over time (e.g., measures with reliable change indices)*

While discussion of each construct is beyond the scope of this article, some of the most salient constructs we recommend considering for inclusion in MBC monitoring for EBPIs are symptom severity, goal attainment, mechanisms of change, and therapeutic alliance. Initially, it is advisable to track the course of the primary presenting concerns that prompted the referral, as symptom reduction is a goal of EBPIs and a common metric of intervention success. Given the high rates of comorbidity between externalising and internalising symptoms in children (affecting approximately 35% of children in the community and 78% referred for clinical services; Polier et al., 2012), combined with evidence that EBPIs reduce both externalising and internalising symptoms (Bloss et al., 2024; Zarakoviti et al., 2021), practitioners may consider using measures that monitor multiple symptom domains to better guide intervention planning and post-intervention needs for families (see Table 1 for measure options).

Although children often present with similar presenting concerns (e.g., emotional and behavioural difficulties), parents may want different outcomes from EBPIs, which are often not captured by symptom screening measures. Therefore, including idiographic measures, such as parent goals, are important to provide a holistic and personalised understanding of progress. Agreement between practitioners and clients about the goals of therapy is a core component of therapeutic alliance (Bordin, 1979) and aligning intervention tasks with client preferences has been linked to decreased rates of drop-out and improved therapeutic outcomes in adult populations (Swift et al., 2018). Since collaborative goal setting is a core component of most EBPIs (van Aar et al., 2021), it should be relatively simple to incorporate these measures into MBC practice. Intervention goals may be focused on parents or the child, and they may be change-based or focused on maintaining stability of desired outcomes (van Aar et al., 2021). Routine goal-monitoring can measure intervention effectiveness and increase client engagement and, subsequently, ensures goals remain a key focus of the intervention.

Assessing variables related to putative mechanisms of change in EBPIs could also potentially enhance the utility of MBC for this population (Scott & Lewis, 2015). Evidence suggests that changes in dysfunctional parenting practices in EBPIs are the mediators (i.e., mechanisms) of change in child behaviour problems (Beelman et al., 2023; Forehand et al., 2014; Laas Sigurethardottir et al., 2025), so monitoring progress in parents’ acquisition and implementation of parenting strategies is important. Monitoring implementation of strategies in EBPIs can be done through parent-report (e.g., completion of homework sheets, discussion about implementation) or direct behavioural observation (e.g., tracking skill acquisition by observing and coding parent-child interactions in session; DPICS, Eyberg et al., 2013). Compared to MBC in adult therapy, utilising behavioural observation is a novel and unique method of routinely tracking intervention progress, and parenting practitioners are encouraged to be creative with how they might incorporate observational outcome measures in their clinical practice.

Finally, in conjunction with measures of intervention progress, it is also recommended that parenting practitioners include measures of common process-related factors, such as therapeutic alliance (Scott & Lewis, 2015). A robust finding in the literature is that therapeutic alliance, commonly referred to as the bond between the practitioner and client, has been positively associated with both child and adult intervention outcomes regardless of therapeutic intervention, such that stronger alliances are associated with better outcomes (Horvath et al., 2011; Karver et al., 2018). Specifically, stronger associations between therapeutic alliance and intervention outcomes have been found for children with externalising disorders than internalising disorders (Shirk & Karver, 2003), and high levels of therapeutic alliance have been associated with greater parenting skills and parental sense of competence in EBPIs (Schmidt et al., 2014). Therefore, measuring therapeutic alliance each session might provide an opportunity for practitioners to alter their behaviours in ways that improve the alliance and subsequently, this may improve parent engagement and intervention outcomes.

In summary, this section has suggested a range of constructs that may be important to routinely measure in EBPIs such as symptoms, goal attainment, implementation of parenting strategies, and therapeutic alliance. While it is recommended that measures are decided prior to starting an intervention, it should be noted that practitioners could include a measure later in an intervention if it becomes apparent that this might be useful (e.g., parent mental health has been identified to interfere with intervention progress). However, the time and resource burden of MBC is a key consideration for EBPIs and not every important domain can be measured. Parenting practitioners may benefit from adopting a needs-led approach to measure selection, such that practitioners have an established battery of brief, psychometrically sound measure options based on the aforementioned factors that can be flexibly and collaboratively tailored to the needs and preferences of the family.

#### Key Consideration 3: Administering Measures

Measures can be administered via pen-and-paper formats, although with the rise of telehealth after the global pandemic, particularly among EBPIs, electronic and digital formats for sending, completing and sharing measures have become increasingly important (de Jong et al., 2025). Computerised tools, known as Measurement Feedback Systems (MFS), have been developed to facilitate MBC through the administration and automatic scoring of outcome measures and immediate provision of feedback reports (see Lyon et al., 2016 for a review). There are several MFS available for children and youth broadly including the Outcome Questionnaire (OQ) system (Lambert, 2015), Partners for Change Outcome Management System (PCOMS; Duncan & Reese, 2015), and the Systematic Therapy Inventory of Change system (STIC; Pinsof et al., 2009). However, there do not appear to be any MFS specifically designed and publicly available for EBPIs (Yu-Lefler et al., 2022).

The frequency of measure administration will depend on the client population, intervention modality, outcomes being tracked, time frame of outcome measures, rate of change anticipated, timing of sessions, and intervention stage (Barber & Resnick, 2022). Since one of the proposed mechanisms of change for MBC is that MBC alerts practitioners to cases requiring attention (e.g., NOT cases) and/or specific areas of concern that may not have otherwise been recognised or attended to (McAleavey et al., 2024), greater frequency of MBC administration might be important early in an intervention to allow for timely adjustments, where required (Barber & Resnick, 2022). For EBPIs, more frequent administration (i.e., session-by-session) is likely needed due to the time-limited and often short-term duration of these interventions. However, the optimal frequency of measure administration might also differ across outcomes measured. For example, we might anticipate that progress toward intervention goals may take more time than other outcome measures (e.g., symptom reduction) and so less frequent administration might be beneficial to increase clinical utility, reduce burden on practitioners and clients, and avoid possible negative effects of clients observing little change in goals early in an EBPI (Låver et al., 2023). As such, decisions about frequency will require clinical judgement and likely some careful experimentation from parenting practitioners about what is most suitable and useful.

### Phase 2: Share

The *Share* phase involves sharing timely feedback with the client about their intervention progress by informing clients about outcome data after each administration, discussing and verifying responses, and building a shared understanding of the data (Barber & Resnick, 2022). A key challenge of applying the *Share* phase to EBPIs is considering how practitioners will manage data from multiple caregivers and integrate multiple perspectives, particularly discrepant perspectives, all of which may have implications for how progress is understood in an EBPI.

#### Key Consideration 4: Integrating Multiple Perspectives

Despite MBC being designed to enhance communication between practitioners and clients, guidance on effective strategies for sharing feedback that motivates behaviour change and improves intervention outcomes remains limited (McLeod et al., 2022 ). Emerging research in adult mental health suggests the impact of MBC depends on delivering feedback in a meaningful client-tailored manner (Brooks-Holliday et al., 2021; Solstad et al., 2021). For example, Solstad et al. (2021) emphasised that no single approach suits all clients, advocating instead for explicit discussion and negotiation between practitioners and clients to align feedback with each client’s therapeutic needs and preferences. This complexity is heightened in EBPIs, where practitioners must consider and reconcile potentially conflicting preferences among multiple caregivers. Consequently, parenting practitioners must demonstrate clinical skill in integrating diverse perspectives, tailoring feedback to be meaningful for each caregiver, and dynamically integrating the feedback within the ongoing intervention process.

When sharing feedback from multiple caregivers, it may be beneficial to incorporate visual representations of the data such as graphs (Brooks-Holliday et al., 2021) and display both parents’ data on the same graph so each perspective can be openly shared and discussed. It is important that the practitioner is not the gatekeeper of the data (Solstad et al., 2021), and instead parents are empowered to take responsibility for their data by bringing their own reflections and interpretations each session. Given that a key goal of EBPIs is to increase self-regulation processes such as self-monitoring, accurate self-reflection, and independent problem solving (Sanders et al., 2019), practitioners should invite parent reflections before discussing their own perspective. This helps to establish the expectation that parents will play an active role in the intervention (Sanders & Lawton, 1993). It is also important to provide simple explanations of the data and tailor the information to individual differences in caregivers’ abilities to process and assimilate information (Sanders & Lawton, 1993). For example, personal and family characteristics that might influence the way practitioners discuss the data include parents’ socio-economic status, ethnic and cultural background, family composition (e.g., one or two parent families, blended families), parents’ level of education, general intellectual functioning, mental health literacy, and preferred learning styles (Sanders & Lawton, 1993). Additionally, clients have been shown to value MBC when discussions include whether measure scores match their experience and practitioners use Socratic questioning to guide what may account for changes in symptoms over time (Brooks-Holliday et al., 2021).

When discussing and integrating feedback from multiple caregivers, it can be helpful to start by focussing on points of agreement in the data before moving to discuss discrepancies. Discrepancies in caregiver perspectives are commonplace, so it is important for practitioners to acknowledge and normalise these differing viewpoints (Tully et al., 2021). Adopting a stance of curiosity is helpful as discrepancies in the data, and parents understanding of them, may offer valuable clinical insight into factors that maintain challenging child behaviour (e.g., conflict over parenting, inconsistencies in parenting), and discussion of discrepancies may influence the course of the intervention in ways that may not have been achievable without this data (Tully et al., 2021). Sharing and discussing discrepancies in a non-judgemental and non-directive way offers a rich opportunity to bring discrepant perspectives into alignment by identifying the source of discrepancies (e.g., differences in conceptualisation of the problem or expectations for child behaviour/parenting responses/intervention), validating each perspective, making sense of the data together, and coming to a shared understanding of how to progress forward. Through these discussions, MBC may increase concordance and communication practices between parents, which has been associated with enhanced EBPI outcomes (Dadds et al., 1987).

### Phase 3: Act

The *Act* phase involves using client feedback to make collaborative decisions about the course of an intervention by evaluating and discussing progress with clients (e.g., improvement, deterioration or no change), brainstorming ideas of how to proceed, and agreeing on a plan of care (Barber & Resnick, 2022). Research shows this phase of MBC is the least utilised in child and youth interventions (Casline et al., 2023), suggesting practitioners may need guidance, especially given the limited support available to inform the clinical practice of tailoring and flexibly implementing child mental health interventions (Georgiadis et al., 2020). For EBPIs, key challenges associated with the *Act* phase include understanding and examining trajectories of change, and the process of using MBC data to optimise clinical decision making and intervention planning.

#### Key Consideration 5: Examining Trajectories of Change in EBPIs

Feedback theory suggests that a discrepancy between the client’s current progress data and their intervention goals or expected trajectory of change motivates the practitioner to adjust the therapy to address this discrepancy (Sapyta et al., 2005). This indicates that practitioners require knowledge and understanding of expected trajectories of client response to therapy. In adult therapy where there is a long history of MBC implementation, statistically derived algorithms are available based on large data sets of client response to therapy. As such, sophisticated Measurement Feedback Systems have been developed that include graphical presentation of progress scores over time with comparison of client scores to a trajectory of normative progress scores, and “alerts” that indicate whether a client is at risk of deterioration or non-response (e.g., OQ system; Lambert, 2015 & PCOMS; Duncan & Reese, 2015). Comparatively, the research exploring trajectories of change in EBPIs is more limited, which prevents the development of similar statistical algorithms of non-response and poses a challenge for practitioners determining whether clients are “off track” for positive intervention outcomes.

Available studies on trajectories of change in EBPIs suggest that symptom reduction is relatively gradual, although it should be noted that there are challenges generalising across EBPIs as there is considerable variation in the duration of differing EBPIs. For example, while many EBPIs are 8 to 12 sessions (Barlow & Coren, 2018), session duration can range anywhere from a single session (Schleider et al., 2017) to more than 20 sessions (Lieneman et al., 2019). In a study exploring a modular cognitive-behavioural intervention for parents of children aged 6–11 years with disruptive behaviour disorder symptoms, Lindhiem et al. (2010) demonstrated that two-thirds of symptom reduction occurred within 10 (60.4% symptom reduction) to 11 (73.1% symptom reduction) sessions. A more recent study of EBPI with parents of 4–7-year-old children with externalising problems demonstrated that 50% of families achieved “optimal outcomes” (score of 0–3 on Behaviour Rating Scale) by 10 sessions, with 75% achieving optimal outcomes within 24–29 sessions, which was the maximum intervention effect (Yu-Lefler et al., 2024). Of note, more consistent attendance was associated with a greater likelihood of reaching optimal intervention outcomes (Yu-Lefler et al., 2024). Within studies on PCIT, four sessions appear to be the minimum dosage required for significant change in caregiver reported child behaviour symptoms (Lieneman et al., 2019) and changes in caregiver skills (Hakman et al., 2009). For example, Lieneman et al. (2019) demonstrated that families who attended at least four sessions of PCIT (regardless of whether they “graduated”) achieved large effect sizes in changes in caregiver-reported child behaviour symptoms from first to last session (*d* = 0.86), whereas families who terminated after attending three or fewer sessions showed very small effect sizes in comparison (*d* = 0.11). Taken together, these results tentatively suggest that families should generally be expected to show a response within 4–10 sessions of an EBPI. However, given the diversity in the format and length of these interventions, it is extremely challenging to make generalisations in change trajectories of EBPIs across differing intervention models. Further research is needed to explore trajectories of change as there is no clear definition of what constitutes a “non-responder” within EBPIs.

In the absence of available datasets to provide statistical algorithms about expected trajectories of change, a more important and reliable factor may be when the key parenting strategies (or ‘core’ components) are delivered and implemented within the EBPI, rather than a set number of sessions. Considering available research, it may be reasonable to expect that change will occur within the first 4–10 sessions, especially if the key components of the EBPI are delivered early in the intervention. As a rough guideline, parenting practitioners should be vigilant to whether changes in MBC data show improvement during this period and if not, consider whether adjustments to the intervention are required. 

Importantly, practitioners need to have a nuanced understanding of how key components of EBPIs might be expected to impact outcome measures. For example, common elements of EBPIs, such as exposure-based tasks or the introduction of consistent limit setting, might be expected to temporarily increase the frequency or intensity of presenting concerns (e.g., anxiety, distress, challenging behaviours) through a phenomenon known as “extinction bursts” (Muething et al., 2024). Understanding such responses is important as, in some cases, a temporary increase in symptom intensity or frequency may be a common trajectory course for certain interventions, while in other cases it can be a cause for concern. 

Further, evaluations should consider trajectory of response over time rather than a single data point (Barber & Resnick, 2022). In EBPI, it is common for parents to present with “bad” weeks, reflected in the temporary worsening of outcome scores, so once-off instances of worsening scores are not generally a cause of concern for practitioners, particularly in the context of temporary life stressors. Discussions with parents about these challenging weeks may reveal external factors that explain challenges experienced, such as illness in the family or changes in routines due to school holidays. Therefore, it is more important for practitioners to consider general patterns in outcome scores over time when determining response to the intervention and be vigilant that worsening of outcome measures is temporary, and that prolonged escalations may instead indicate that an adjustment to the intervention is required.

A related challenge for practitioners is the interpretation of longitudinal clinical data in the context of decisions about intervention progress. While statistical methods, such as Reliable Change Indices (Jacobson & Truax, 1991), are often utilised in research to determine whether change across interventions constitutes meaningful improvement, it is unrealistic and impractical to expect practitioners to use these methods in clinical practice. In the absence of Measurement Feedback Systems with statistical algorithms outlining expected trajectories of change, practitioners might consider other indices of response, such as whether progress scores have reduced to below the clinical cut-off on measures, the percentage of reduction in symptoms from baseline, or whether the reported changes have been functionally meaningful for the child and family (e.g., if child externalising symptoms have reduced but the child continues to have no friends or is unable to participate in social activities, the intervention might have only been partially successful). Ultimately, data indicating ‘response’ and ‘non-response’ should also factor in whether the family perceives a meaningful improvement in their child’s presentation. While the above methods are viable options, the development of a Measurement Feedback System that provides expected trajectories of change across EBPIs would be an important resource for the use of MBC in EBPIs.

#### Key Consideration 6: Using MBC Data To Guide EBPI Decisions

A primary goal of MBC is to leverage MBC data to inform and enhance clinical decision-making about intervention planning. Practitioners must be able to interpret and understand the MBC data to identify NOT cases, and then sensitively and openly discuss this lack of positive progress with parents, a process that many practitioners will find uncomfortable (de Jong et al., 2023). Ultimately, practitioners need to be able to identify and manage the factors impeding intervention progress, resulting in a series of clinical decisions and actions (de Jong et al., 2023). Clinical decision making needs to be intentional and dynamic, that is hypothesis-driven and responsive to client needs, and practitioners should have an evidence-based understanding of the intended consequences of intervention decisions (Georgiadis et al., 2020). Practitioners also need to be able to provide a clear rationale to parents about how each modification to an intervention will address their needs, thereby allowing parents to meaningfully participate in shared decision making around their intervention plan (Barber & Resnick, 2022). In fact, research has demonstrated consensus among expert practitioners that this type of formulation driven practice is a core practitioner competency for delivering EBPIs, especially with complex presentations (Barker & Hawes, 2024). Consequently, using MBC data in the context of an ongoing intervention to inform clinical decision making is an inherently complex clinical skill that requires guidance, training and supervision. 

**Fig. 1:**
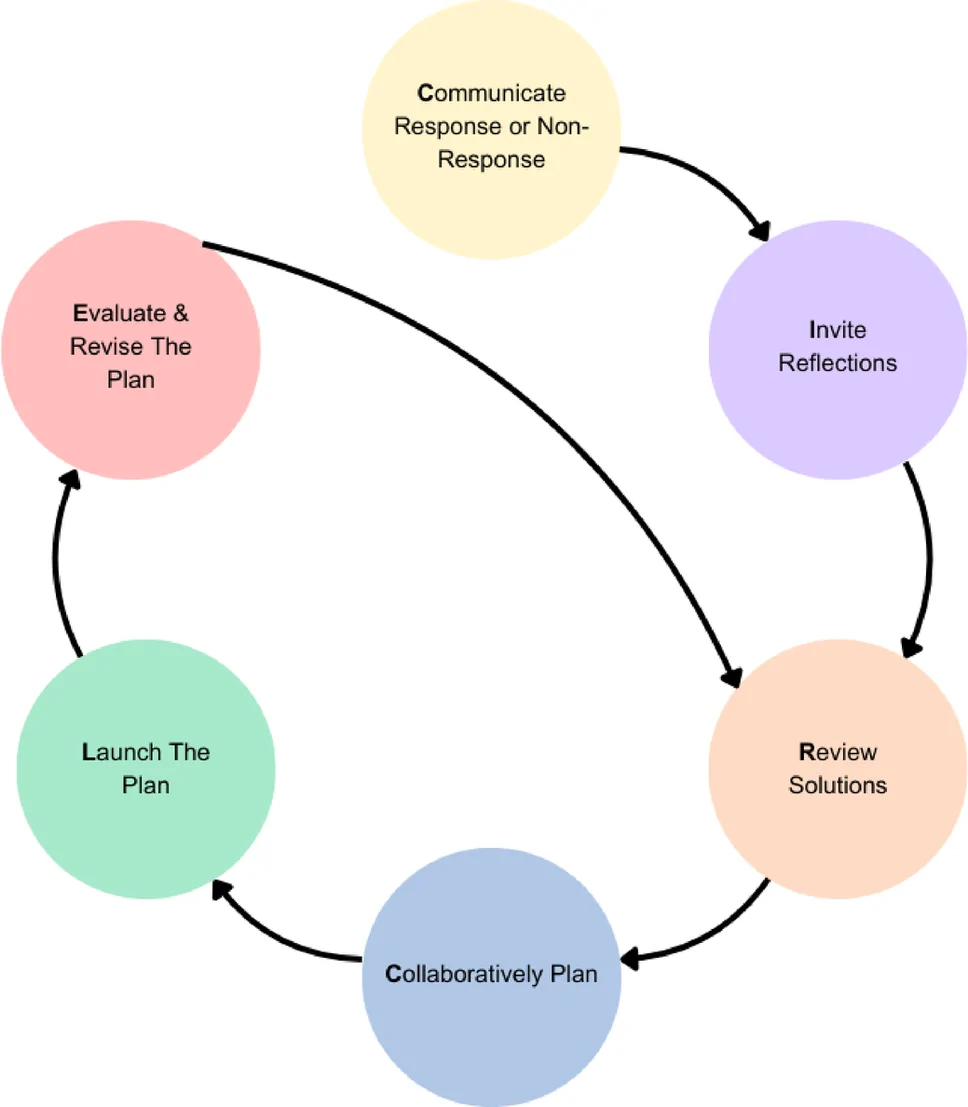
The Parenting CIRCLE of MBC, A Framework for the Act Phase of MBC in EBPIs

As a starting point, we propose the “*Parenting CIRCLE of MBC”* (see Fig. 1), a six-step guide designed to support the *Act* phase of MBC. This guide applies a generic evidence-based framework of “clinical troubleshooting” (de Jong et al., 2023) to EBPIs, to inform clinical decision making and help structure feedback discussions with families. Although the *CIRCLE* framework is focused on the *Act* phase, MBC operates as a continuous feedback loop between practitioners and clients, with the *Collect*,* Share*, and *Act* components recurring and often overlapping throughout the intervention. The *CIRCLE* framework outlines key considerations for dynamically tailoring EBPIs to parent preferences and needs using data- and formulation-driven practice. It provides guidance on communication style and language, relevant prognostic factors, strategies for engaging parents in collaborative problem-solving, possible intervention options, and methods for monitoring and evaluating intervention adjustments. We also present a case description that demonstrates the *Collect*,* Share*,* Act* model in an EBPI (see Textbox 1).

##### The “Parenting CIRCLE of MBC”

1. *“Communicate Response or Non-Response”.*

The first step is to evaluate the MBC data to determine the family’s response to the intervention, specifically whether the family is responding (i.e., has seen improvements and is “on track”) or not responding (i.e., has seen no change or a worsening of outcomes and is “off track”) to the intervention (Barber & Resnick, 2022). This involves the practitioner and family using MBC data, as well as other relevant sources of information not represented by the data (e.g., contextual factors, constructs not measured in MBC), to come to an understanding of the family’s progress (Barber & Resnick, 2022). In the absence of adequate Measurement Feedback Systems and known expected trajectories of change in EBPIs, practitioners might consider indices of response, such as whether the child scores have reduced to below the clinical cut-off on measures. To determine whether a family is responding (or not) to the intervention, practitioners will need enough data points to be able to see a pattern over time. This means that these types of conversations with clients will not typically occur until at least 3–4 data points have been collected across the intervention.

Once the trajectory of the family has been identified, this needs to be communicated to the family. It is the responsibility of the practitioner to start this conversation and create a safe and therapeutic space to explore any concerns that may be occurring with the family’s response to the EBPI (Hawes & Dadds, 2021). Starting these conversations establishes this as a shared issue between the practitioner and family that be openly explored, understood and problem-solved together (Hawes & Dadds, 2021). It is recommended that practitioners avoid jargonistic and pessimistic terms such as ‘non-response’ and instead use lay terms such as ‘on-track’ and ‘off-track’ or simply describe what the data is showing (e.g., an increase in aggressive behaviour). Relatedly, when discussing intervention non-response, it can be helpful to normalise the struggle of the change process to ensure that families do not feel at fault or blamed (Hawes & Dadds, 2021).

Importantly, practitioners should check whether the MBC data is consistent with the parents’ own sense of intervention progress (Barber & Resnick, 2022). Sometimes MBC data can indicate that a family has not experienced change from an EBPI; however, it may just be that the measures have not captured the meaningful change that has occurred for the family. Any discrepancies between the MBC data and parent report should be discussed.

For example, *“Looking at these graphs over the past week few weeks*,* I’m noticing that [your child’s] aggressive behaviour seems to be getting more frequent. Oftentimes when the program is working*,* parents know it early on and can see positive differences in behaviour. I’m wondering if you both feel that right now?” …*or*… “If we look at these progress charts over the past few weeks*,* it doesn’t look like the graphs are changing. In my experience working with families*,* when we don’t see change it can be an important signal that we might have missed something valuable. Does that fit with your experience?”*

2. *“Invite reflections”.*

The aim of this step is to use the MBC data to encourage parent self-reflection, with a focus on building insight and understanding into the change processes that may be occurring or not occurring throughout the course of the EBPI. Practitioners should invite parents to share their reflections and offer ideas about relevant factors that may be influencing the family’s progress. By inviting parents to reflect on their outcome data first, parents might raise other areas of improvement or concern that have not been directly tracked with the measures but are important to the intervention process (e.g., social supports, relationship with school, etc.).

For cases with clinical improvement across the course of the EBPI, it is still important for practitioners to regularly review and discuss data each session with parents. In line with a “self-regulation approach” to EBPI, these discussions should be used to facilitate parents’ ownership over these change processes (Sanders et al., 2019). Parents commonly attribute changes to a range of external factors such as chance, age, maturational factors, or practitioners’ skills. Therefore, it is recommended that these discussions encourage parents to attribute improvements in their family to either their own or their child’s efforts (Sanders et al., 2019). To do this, practitioners might ask parents to identify what they are doing differently that has enabled the child’s behaviour to change (Sanders et al., 2019).

For example, practitioners might enquire: *“It’s wonderful to see that things seem to be improving at home*,* and that [your child] is cooperating more often when you ask him to do things. What are you both doing differently since you started the program that has helped you see these positive changes?”*

Conversely, for cases with deterioration or non-response to the EBPI, it is likely that some form of adjustment to the intervention plan is required. For these cases, the aim is to identify relevant factors that might be impeding intervention progress. It is important for practitioners to approach this step with an attitude of curiosity, remaining open to multiple contributing factors, and willing to discuss their initial assumptions if needed (de Jong et al., 2023).

For example, practitioners might ask: *“When we’re not seeing change*,* it can help to try to work out what might be getting in the way and stop a parenting program like this from working. What do you think might be happening?”.*

Identifying the reasons for non-response to an EBPI is inherently complex, as it may involve several factors, some of which might not be modifiable (de Jong et al., 2023). As de Jong et al (2023) recommend, this step should involve practitioners guiding parents to consider prognostic factors, including contextual, risk, and process factors, that might be contributing to non-response. A list of relevant risk factors that might be considered for EBPIs are listed in Table 2.

**Table 2:** Contextual, process and personal risk factors for EBPIs

Contextual factors	Process factors	Individual/Personal factors
• Parent mental health and wellbeing. • Interparental conflict. • Socio-economic factors. • Cultural beliefs. • Life stressors (e.g., work stressors, death of a family member).	• Poor alliance or alliance ruptures. • Engagement problems. • Motivation problems and readiness for change. • Attendance issues (e.g., only one parent participating). • Low adherence to between-session homework tasks. • Resistance to an intervention.	• Severity and pervasiveness of child problems. • Developmental factors (e.g., developmental delays, temperamental features). • Comorbidities in presentation (e.g., internalising problems, neurodiversity). • Quality of parenting. • Parent attributions/beliefs (e.g., stable child-based attributions).

A key part of this step is to go beyond just identifying possible factors that are interfering with the intervention and begin to generate hypotheses about *how* these factors might interfere for this family, ultimately coming up with a unique formulation for each family (de Jong et al., 2023). This will help to guide appropriate solutions and requires practitioners to use clinical judgement, empirical research, and possibly even input from colleagues and clinical supervisors. There are a number of important questions to consider including: (1) How might these factors impact parent goals? (2) How might these factors impact change mechanisms? That is, how might these factors interfere with parenting practices or implementation of new strategies? How might they impact parent-child interactions? (3) How might these factors influence a parent’s capacity to cope? (4) Do these factors represent a skill deficit (e.g., parents have not yet acquired a skill) or performance deficit (e.g., a skill exists but it not yet being applied effectively)? (5) How might these factors interact together? (de Jong et al., 2023).

3. *“Review Solutions”.*

Once relevant factors and pathways for intervention interference have been identified, practitioners must consider if adjustments to the intervention are needed. If so, the next step is to formulate and discuss some solutions to target these factors. This step involves collaboratively brainstorming and reviewing identified solutions with the parents that will allow progression forward (Barber & Resnick, 2022). Practitioners are recommended to model and scaffold this process but ultimately empower parents to draw on their own expertise to identify solutions (Hawes & Dadds, 2021). As with any structured problem-solving process, it is recommended that all possible solutions are noted and discussed, regardless of how realistic or feasible they may be. This will allow parents to feel heard and for their perspective to be valued, which should help with their “buy in” to try an agreed-upon solution. Solutions might focus on increasing factors that facilitate change, reducing barriers to change, or identifying available supports/resources.

Broadly, possible solutions in EBPIs might include: adjusting intervention goals; adjusting the pace of the intervention; adjusting the intervention intensity; making adjustments to the intervention team; setting homework tasks to address concerns; omitting intervention content; addressing interfering problems; targeting secondary problems; adjusting the order of intervention content; initiating a referral for additional forms of care; liaising with other professionals; changing intervention modalities; and terminating or discharging. If MBC data is on-track and improvements are being observed, the obvious solution may be to proceed as planned without making any unnecessary adjustments.

4. *“Collaboratively Plan”*.

This step involves the practitioner and parents weighing up the pros/cons of each proposed solution to ultimately guide a course of action. Collaborative decisions around appropriate actions should be guided by a combination of data, parent experiences and beliefs, practitioner judgement, and supervisor input where appropriate. Practitioners should have an evidence-based understanding of the intended consequences of intervention decisions (e.g., they should consider short- and long-term goals of an intended intervention modification; Georgiadis et al., 2020), and they should be able to provide a clear rationale to parents so they understand how each modification will address their needs (Barber & Resnick, 2022). Given that each course of action is based on a set of hypotheses about what might be driving non-response, the plan could be pitched as an “experiment” to be trialled and tested, where appropriate (de Jong et al., 2023). As such, there should be a clear and observable goal and time frame established for a review of whether the plan was effective. An exception to this would be if the plan was to terminate the intervention.

In the context of improvements, intervention decisions are likely to involve a simple set of “actions”, including continuation of the intervention as originally planned; incorporation of additional intervention components to target secondary factors that have emerged across the course of the intervention (e.g., changing parent goals as initial goals have been achieved); and termination of the intervention. It is particularly important for practitioners to be guided by parent progress toward intervention goals as these idiographic measures often provide a good roadmap for each of these actions.

5. *“Launch the plan”.*

Once a plan has been collaboratively generated and agreed upon, the plan should be implemented. The plan should be clear, and parents should understand what is expected of them and how this plan will address their needs (Barber & Resnick, 2022). Practitioners should monitor implementation and be ready to facilitate the evaluation process with the parents.

6. *“Evaluate & Revise the plan”.*

Following the principles of MBC, the data should be used to determine if the plan has been successful. If symptoms are improving and the family are reporting progress toward goals, a positive home life, and more confidence, then it is evident the plan has worked. However, if the outcome data has remained unchanged or has worsened during or after the plan has been implemented, it is evident that the plan has not worked and needs to be revised (de Jong et al., 2023).

If the family has started to improve and progress forward in the intervention, then several actions might be indicated, including resuming the original intervention plan; offering a modified intervention plan; or terminating if adequate progress has been made (de Jong et al., 2023). However, if the family’s progress remains unchanged or has worsened, a different set of actions might be indicated, including: refining the plan identified in step 4 to address any obstacles for progress; trialling one of the other solutions identified in step 3; identifying other factors impeding progress; that is, starting the process again from step 2; or terminating the intervention and referring the family to an alternative intervention or service (de Jong et al., 2023). Regardless of the actions taken, the final step in the *CIRCLE* framework should involve resetting the shared therapeutic agenda with the parents to ensure the therapeutic team is on the same page.

## Other Considerations

### Key Consideration 8: Considering Organisational Support for MBC Implementation

The *Collect*,* Share*,* Act* model primarily focuses on implementation factors at the practitioner level, which is only one part of an implementation science framework. Practitioners must also consider contextual and organisational factors that may constrain implementation of MBC and therefore impact adoption and sustainability of MBC for EBPIs over time (Lewis et al., 2019; Youn et al., 2024). Contextually, EBPIs are typically delivered in community settings as part of routine practice, predominantly within mental health or child welfare organisations, but also within social services, clinics, schools, non-profit organisations, and churches (Pinto et al., 2024). While evidence-based assessment is fundamental to the training programs of certain professions (e.g., psychologists), EBPIs are delivered by a range of practitioners with varied educational backgrounds including social workers, nurses, psychologists, counsellors and educators (Pinto et al., 2024). With such diverse settings and practitioners, each clinic and/or organisation will need to develop a tailored approach that enables meaningful integration and training of MBC that is consistent with the organisational culture, values, resources and goals of local implementation sites (Lewis et al., 2019).

Two key organisational factors that should be considered when integrating MBC into EBPIs include ensuring sufficient leadership support and available resources (Mackrill & Sorensen, 2019; Lewis et al., 2019). Leadership support is essential for any successful organisational change, and without strong leadership support, it is unlikely that MBC will be successfully adopted and integrated into clinical practice by parenting practitioners. For example, the leadership team should view MBC as a valuable enhancement to EBPIs and promote an organisational culture that recognises client feedback as a central component to improve service delivery and outcomes (Lewis et al., 2019; Mackrill & Sorensen,^2019^). Additionally, the literature consistently highlights the importance of a dedicated individual (e.g., “MBC Champion”) or team that is responsible for driving and overseeing MBC implementation within an organisation, with the aim of identifying and resolving any issues that arise during the implementation process, as well as coordinating training and supervision of staff to build MBC competencies within an organisation (Lewis et al., 2019; Mackrill & Sorensen,^2019^). It is important that implementation teams are composed of stakeholders from diverse leadership levels (e.g., managers, front-line practitioners, administration staff), who have received appropriate MBC training to enable them to understand and use MBC data to inform decisions that drive service quality, and who have the influence and power to make organisational changes (Mackrill & Sorensen, 2019). Strong leadership support should help to improve practitioner attitudes toward MBC practices and may mitigate concerns about the potential misuse of MBC data to evaluate practitioner competence and inform renumeration and advancement of practitioners (Mackrill & Sorensen, 2019). However, even with strong leadership support, policies that require use of MBC will likely be needed to motivate staff to adopt MBC, although integration of such policies should be gradual to allow staff time to adjust to a change in practice (Mackrill & Sorensen, 2019).

Second, organisations need to consider the availability of resources required to implement MBC. A key leadership task is to ensure access to valid and clinically useful measures and the technology infrastructures to administer, score and graph these measures (e.g., MFS), as well as adequate MBC training programs (Lewis et al., 2019; Mackrill & Sorensen, 2019). Given the high turnover rates of staff trained in MBC, organisations need to also consider availability of *ongoing* training programs, specifically ongoing supervision and consultation to support sustained implementation (Lewis et al., 2019). In essence, effective implementation at an organisational level is complex and requires significant planning and coordination of staff and resources. Of note, Dollar et al (2020) have developed a 13 step *“MBC Implementation Planning Guide”* that can be used by local implementation teams to develop an MBC program tailored to each unique context. It is recommended that practitioners consider using this implementation guide within their organisational setting as without sufficient organisational readiness and committed leadership support, attempts to integrate MBC into EBPIs are unlikely to succeed.

### Key Consideration 9: Utilising MBC with Culturally and Linguistically Diverse Populations

There is general consensus that consideration of contextual and cultural factors is a priority in the implementation of evidence-based mental health interventions, particularly given consistent documentation of disparities in mental health interventions for culturally and linguistically diverse (CALD) populations. Among EBPIs grounded in social learning theory, evidence supporting transportability of these interventions is mixed. Some meta-analyses demonstrate comparable effect sizes when EBPIs are implemented outside their country of origin (Gardner et al., 2016), with no significant differences between EBPIs that have been transported internationally or locally developed (Leijten et al., 2016). These findings suggest the core principles of EBPIs are broadly applicable and transferrable across cultures, and that cultural adaptation may not always be necessary. However, a recent large scale meta-analysis (279 RCTs from 33 countries) demonstrated that ethnicity moderated EBPI outcomes, such that trials with predominantly racial/ethnic minority families showed smaller improvements in negative parenting and fewer reductions in child externalising behaviour outcomes, compared to trials with mostly majority racial/ethnic families (Backhaus et al., 2023). CALD families also face disproportionately higher barriers to EBPI access and engagement, including socioeconomic disadvantage, parenting stressors, and negative perceptions of the relevance of EBPIs (Kazdin et al., 1995; Lavigne et al., 2010; McCabe et al., 2020). Consequently, CALD families have been shown to experience lower enrolment rates, longer completion times, and higher drop-out rates for EBPIs, compared to majority white families (Lavigne et al., 2010; McCabe et al., 2020; Pina et al., 2019). Taken together, there is a need for interventions to maximise enrolment, attendance and retention of CALD populations, without undermining the efficacy of the original intervention, and MBC might offer promise for these populations.

MBC has recently been positioned as an engagement intervention with the potential to reduce mental health disparities and promote better quality care among CALD populations through four distinct but overlapping pathways (Barber et al., 2025). Specifically, MBC has been theorised to: (1) optimise treatment quality via personalised treatment planning, early detection of treatment non-responders, and proactive and timely treatment adjustments; (2) promote mental health literacy through feedback conversations that facilitate shared understanding of mental health among practitioners and clients; (3) improve therapeutic alliance via enhanced client-centred communication and collaborative decision-making; and, (4) increase transparency about treatment progress and clinician accountability to improve client satisfaction and treatment retention (Barber et al., 2025). As a method that facilitates the adaptation of mental health interventions to the needs, values and preferences of clients, MBC may be a way to maximise enrolment, attendance, and retention of CALD populations, without undermining the established efficacy of original mental health interventions. However, despite MBC’s promise as an engagement intervention, it is unclear whether MBC is sufficient it its standard form to address mental healthcare disparities among CALD populations, or whether modifications are required to optimise the cultural appropriateness of MBC (Barber et al., 2025).

Concerns have been raised about the use of standardised, nomothetic outcome measures for CALD populations that have not been tested and validated among multiple racial/ethnic groups (Jacob & Edbrooke-Childs, 2022). As such, further development may be required to establish psychometrically valid and reliable outcome measures for diverse populations that are both sensitive to change over time and that are normed for populations of interest. In the absence of culturally sensitive standardised measures, it has been suggested that practitioners augment standardised assessments with idiographic measures to capture personalised progress among CALD populations (Barber et al., 2025; Connors et al., 2023; Arora et al., 2025). Some researchers have also suggested that clinicians consider incorporating sociocultural measures of perceived discrimination, racial identity or other constructs related to race, ethnicity, nationality or cultural identity (Connors et al., 2023). Sociocultural measures could prompt open communication with clients about whether, and how, ethnicity and culture influence their beliefs, expectations, goals or preferences about treatment, and encourage these conversations to be a continued part of treatment, where helpful and relevant (Connors et al., 2023).

Additionally, researchers have argued that standard MBC does not adequately target common perceptual barriers to mental health interventions experienced by CALD populations, such as negative beliefs about treatment and poor therapeutic alliance (Connors et al., 2023; Arora et al., 2025), and that MBC does not replace the need for clinicians to have foundational competencies in culturally-responsive and safe mental health care (Barber et al., 2025; Connors et al., 2023). As such, culturally-tailored versions of MBC have been proposed to optimise MBC’s relevance, acceptability and effectiveness among CALD populations. For example, the Strategic Treatment Assessment with Youth (STAY) model has been developed as a culturally tailored version of the *Collect*,* Share*,* Act* model of MBC for CALD adolescents (Connors et al., 2023; Arora et al., 2025). The STAY model comprises three modifications, including the addition of an initial phase, *Introduce*, focused on providing a client-centred rationale for MBC and explicit assessment and discussion of treatment expectations; the incorporation of measures of therapeutic alliance and individualised treatment goals that draw upon cultural strengths and culture-specific protective factors; and the inclusion of cultural competence training for clinicians (Arora et al., 2025).

While there is broad consensus that contextual and cultural factors must not be overlooked when implementing evidence-based mental health interventions, it is unclear the extent to which MBC needs to be adapted to fit CALD populations. Research is needed to determine whether standard MBC can optimise engagement and outcomes for CALD populations, and whether race and ethnicity moderate mechanisms and proximal outcomes in MBC (Barber et al., 2025). Research is also needed to establish whether culturally modified MBC outperforms standard MBC that has been applied flexibly and sensitively within a culturally competent framework (Lau, 2006).

## Research Agenda & Recommendations for Clinical Practice

MBC presents an exciting and promising approach to target issues of non-response and drop-out and improve outcomes in EBPIs. Given the effects of MBC are *additive* to the standard effects of psychological interventions, MBC could be a relatively simple intervention to bolster the stagnating effect sizes of EBPIs without requiring significant modification to existing clinical practice. MBC is not a replacement, but rather a complement, to clinical judgement allowing parenting practitioners to thoughtfully capture and integrate parent outcome data into EBPIs. MBC provides a vehicle for person-centred care by increasing communication and collaboration between practitioners and parents and providing parents with a sense of agency by engaging parents in shared decision making about their care. While integrating MBC into existing interventions appears seemingly straightforward, the generally low implementation rates among both research and community settings indicate that implementation of MBC is challenging, nuanced, and requires careful consideration. This paper has demonstrated that generalising implementation practices from adult individual therapy settings to EBPIs is not sufficient, and implementation of MBC in EBPIs represents an important and complex gap given the inclusion of multiple caregivers in these interventions. While extant reviews exist outlining the evidence and implementation considerations of MBC broadly, this paper argues that an overlooked barrier to MBC implementation is the knowledge and skills needed to implement MBC, and subsequently the need for guidance and training in the application of MBC to specific therapy modalities, such as EBPIs.

This paper has used the *Collect*,* Share*,* Act* model (Barber & Resnick, 2022) to explore how MBC can be operationalised in EBPIs, outlining key challenges and critical areas of consideration for implementation across the three core phases of MBC—*collecting* routine feedback from parents, *sharing* and discussing feedback with parents, and *using* parent feedback to guide collaborative decision making and signal when intervention adjustment is needed. The *Parenting CIRCLE of MBC* has been introduced with a case example to illustrate a framework for how to implement the *Act* phase of *Collect*,* Share*,* Act* Model, and how MBC data can support clinical decision making in EBPIs according to client progress. While we hope this paper represents a first step in supporting practitioners to integrate MBC in EBPIs, we also recognise that future research is needed to build an evidence-base for MBC in EBPIs.

To guide future investigation of MBC in EBPIs, this paper proposes a research agenda focused on: (1) investigating the efficacy of MBC to reduce drop-out and improve EBPI outcomes through high-quality research designs, such as gold-standard RCTs and adaptive treatment frameworks such as Sequential Multiple Assignment Randomised Trials (SMART), which are multistage randomised trial designs that allow subjects to be randomised multiple times in a sequential manner, according to data-driven decisions at selected critical time points (Almirall & Chronis-Tuscano, 2016); (2) investigating whether MBC increases father engagement and satisfaction; (3) evaluating whether nomothetic or idiographic measures are associated with better intervention outcomes, and practitioner and client satisfaction; (4) testing putative mechanisms of change in MBC; (5) identifying rates of evidence-based assessment and MBC use among parenting practitioners globally and studying the barriers and facilitators to implementing MBC; (6) considering the use of technology infrastructures (e.g., Measurement Feedback Systems) to support MBC in EBPIs that enable the integration of multi-informant data; (7) developing expected trajectories of change across EBPIs and algorithms that facilitate the use of MBC data to identify “on-track” and “off-track” families; (8) evaluating the *Parenting CIRCLE of MBC* as a framework to enhance engagement and satisfaction from families, and reduce rates of drop-out from EBPIs; (9) developing and evaluating MBC training programs and supervision practices to build practitioner MBC competencies, and making these freely available; and, (10) examining factors that influence organisational support for MBC implementation in EBPIs, and practices to improve organisational supports.

Despite extant literature investigating the efficacy of MBC over the last decade, the application of MBC to EBPIs remains a significant gap in the field. This paper builds on the established evidence based for MBC by highlighting key challenges and considerations for the implementation of MBC in EBPIs, through the *Collect*,* Share*,* Act* framework. This work has particularly highlighted that MBC is both a clinical intervention and clinical skill that requires considerable planning and tailoring for specific modalities, populations and settings.

**Textbox 1:** Case example of the application of the collect share act model for an EBPI

The mother of a 5-year-old boy was referred for an EBPI for concerns with frequent and protracted temper tantrums, non-compliance with parent instructions, physical aggression toward parents and sibling, impulsivity and high energy levels. Her main concern was the impact of these challenges on family functioning, particularly length of time to complete daily morning and evening routines, the fatigue and distress associated with parenting, and the impact of these difficulties on the marital relationship. * Collect*: The process of MBC was discussed with a rationale provided for monitoring progress over time. Due to busy schedules, the mother’s preference was that she would participate in the EBPI by herself and would discuss the strategies independently with her husband. Given the presenting concern involved externalising behaviours with symptoms of ADHD, it was collaboratively decided to use a broad symptom measure, the Pediatric Symptom Checklist (PSC-17), as well as measuring program goals and implementation of program strategies. To start, the PSC-17 externalising score was 12/14 (with a clinical cut-off score of 7), the attention score was 7/10 (with a clinical cut-off score of 7), and the internalising score was 2/10 (with a clinical cut-off score of 5). The practitioner implemented a brief EBPI, which aimed to enhance the parent-child relationship by increasing positive reinforcement of desirable child behaviour and strengthening the parent-child relationship through child-centred play, followed by positive discipline strategies focused on setting clear family rules, providing effective instructions, and use of calm, consistent and non-violent consequences to oppositional and aggressive behaviours. * Share*: After three sessions, the externalising score on the PSC-17 had dropped to 8/14 demonstrating improvements in the targeted behaviours. The mother agreed that these scores matched her perception of the family’s response to the program and noted more positive moments in her relationship with her son. Factors that might have contributed to the positive changes were discussed. The mother attributed the changes mostly to external factors such as the consistent school routine and the child maturing but after discussion with the practitioner, was able to attribute some of that change to her own efforts and consistent change in parenting responses. *Act*: Given the positive trajectory so far, it was agreed to continue with the current intervention plan. However, at the sixth visit, the externalising score had increased to 10/14 representing a gradual trajectory of worsening since the third session. *Share*: These results were discussed and the mother confirmed that the scores reflected a worsening of behaviour at home, most notably arguments between the child and his father, as well as more frequent temper tantrums in response to parent demands. *Act*: Given the worsening trajectory of change, the practitioner communicated that these results might be a signal that the care team had missed something valuable. Through exploration with the mother around contextual and risk factors, she disclosed that the child’s father disagreed with the parenting strategies in the intervention and refused to implement the strategies, which led to lengthy arguments – sometimes in front of the children. The practitioner hypothesised that the father’s disagreement may have been undermining intervention progress due to inconsistency in parenting practices across the parenting team, conflict between the parenting team, and that change factors were inadvertently being modified in only one part of the parental subsystem. Fortunately, many of the factors identified were modifiable through intervention (e.g., interparental conflict, inconsistency in parenting team, negative attitudes toward the EBPI). The practitioner and mother discussed whether the intervention needed to be adjusted. Several possible options were discussed, including pros/cons of each option, and the mother ultimately decided that she wanted her husband to participate in the EBPI with her. A plan was formulated to invite the father into the “care team”. Following best practice principles for father engagement (Lechowicz et al., 2019), the practitioner contacted the father, discussed his importance in the care team and the need for his perspective, and invited him to the next session. The father agreed to attend the next session. The practitioner and mother collaboratively decided to continue with the EBPI with the father participating, and to monitor progress over the next 2–3 sessions. * Collect*: Given that the father was engaging in the EBPI, the practitioner reviewed the rationale for MBC and administered the PSC-17 with both parents to gather a new baseline. *Share*: The practitioner graphed both parents’ data and shared the graphs together, the practitioner facilitated the conversation by asking the parents to reflect on their own data and how this was similar/different from their partner. After three sessions with the father, scores on the PSC-17 had started to decrease for both parents. *Act*: The family and practitioner collaboratively decided to continue with the EBPI and continue to monitor progress.	

Case example is based on a hypothetical scenario

## Data Availability

No datasets were generated or analysed during the current study.
